# Detecting the interferences in adrenocorticotropic hormone measurement - three cases reinforcing the efficiency of the complementary clinical and laboratory audit

**DOI:** 10.11613/BM.2024.010802

**Published:** 2023-12-15

**Authors:** Tugba Barlas, Mehmet Muhittin Yalcin, Zakir Osmanov, Ozlem Gulbahar, Alev Eroglu Altinova, Mujde Akturk, Mehmet Ayhan Karakoc, Ilhan Yetkin, Fusun Balos Toruner

**Affiliations:** 1 Department of Endocrinology and Metabolism, Faculty of Medicine, Gazi University, Ankara, Turkey; 2 Department of Biochemistry, Faculty of Medicine, Gazi University, Ankara, Turkey

**Keywords:** adrenal insufficiency, adrenocorticotropic hormone, Cushing’s syndrome, immunoassay, interference

## Abstract

Accurate measurement of adrenocorticotropic hormone (ACTH) is crucial in the evaluation of pituitary and adrenal disorders. Although great progress has been achieved in ACTH measurement with immunometric assays, interference may occur and adversely affect the clinical management. The report contributes to compiling the evidence on the clinical challenges with the management of the interferences in the ACTH measurement by presenting three cases: two with clinically overt hypercortisolism and discrepant ACTH concentrations within the reference interval; the third case describes the falsely elevated ACTH in a patient with secondary adrenal insufficiency. In all patients, the results obtained with the two immunometric platforms, chemiluminescence (CLIA) immunoassay (Siemens, Immulite) and electrochemiluminescence (ECLIA) immunoassay (Roche, Cobas), were discordant. Serial dilution of plasma samples revealed nonlinearity. After polyethylene glycol (PEG) precipitation recoveries were less than 22%, 26%, and 3%, respectively, supporting interference. Moreover, a decrease in ACTH concentration after incubation in a heterophile antibody-blocking tube was observed in the second case. In the first case, misinterpretation of ACTH led to inferior petrosal sinus sampling (IPSS), whereas timely detection of assay interference prevented further investigations in other cases. Increasing awareness regarding ACTH interference and comprehensive approach in evaluation could allow timely detection, helping to prevent unnecessary testing and perplexing clinical outcomes.

## Highlights

Comparing test results to clinical data enhances the identification of interferences

Misinterpretation of ACTH as a result of interference may lead to unnecessary testing

Despite the interference, ACTH concentration can be in the reference interval

## Introduction

The diagnosis and differential diagnosis of Cushing’s syndrome (CS) might be challenging in clinical practice. The accurate measurement of plasma adrenocorticotropic hormone (ACTH) has an important role in the differential diagnosis of CS in addition to the monitoring of hypothalamic-pituitary-adrenal (HPA) axis disorders, including adrenal insufficiency and congenital adrenal hyperplasia (*1*, *2*). Although significant progress has been achieved in measuring ACTH with two-site “sandwich” immunometric assays, analytical interferences may rarely occur as a result of lipemia and the presence of heterophile antibodies, hormone fragments, or precursors (*3*, *4*). Heterophile antibodies are a heterogeneous group of antibodies that can include autoantibodies, therapeutic immunoglobulins, anti-animal antibodies, or rheumatoid factor. Their common feature is the ability to cause interferences in immunometric assays by interacting with the reagent antibodies (*3*, *4*). Unrecognized ACTH assay interference may have a negative impact in diagnosis. Although the number of reported cases of the interferences in measuring ACTH concentration is not high, the associated advert outcomes in clinical management can be significant (*2*, *3*, *5*-*9*). We aimed to report three cases of ACTH interference and contribute to compiling the evidence on the clinical challenges due to the interference in plasma ACTH measurement and laboratory tools to manage them.

## Laboratory analyses

Table 1 contains the relevant clinical, imaging, and laboratory data.

**Table 1:** Clinical, imaging and laboratory findings with reference intervals of presented cases

**Case**	**1**	**2**	**3**
**Signs and symptoms**	Weight gain, hair loss, irritability, fatigue, easy bruisability	Weight gain, abdominal striae, moon face, buffalo hump, menstrual irregularities	None
**Imaging (MRI)**	Pituitary: microadenoma Adrenal: unilateral adenoma	Pituitary: no adenoma Adrenal: unilateral adenoma	Pituitary: heterogeneous contrast, no adenoma Adrenal: n/p
**Cortisol concentration, nmol/L, (reference range)** (Dxl 800 Beckman Coulter)			
Basal morning (08 a.m.), (184-623 nmol/L)	565	1597	195
Late night (11 p.m.), (0-276 nmol/L)	546	1007	n/p
Morning (08 a.m.) after 1 mg DST	557	789	n/p
24-h urinary, (0.159-1.108) µmol/24-h	0.709	1.667	n/p
DHEA-S, (0.9-11.1) µmol/L	0.2	0.3	n/p
**ACTH concentration, pmol/L, method (reference range)**			
CLIA (Siemens Healthcare Diagnostics Inc. NJ, USA) (0-10 pmol/L)	9.3	8.5	73.5
ECLIA (Roche Diagnostics GmbH, Mannheim, Germany) (1.6-13.9 pmol/L)	< 0.3	< 0.3	4.8
After serial dilution CLIA (Siemens Healthcare Diagnostics Inc. NJ, USA) (0-10 pmol/L)	½ - 10.4 ¼ - 30.4 ⅛ - 35.3	½ - 11.9 ¼ - 19.9 ⅛ - 20.9	½ - 38.6 ¼ - 19.6 ⅛ - 16.1
After heterophile blocking tubes CLIA (Siemens Healthcare Diagnostics Inc. NJ, USA) (0-10 pmol/L)	8.4	2.1	69.3
Recovery (%) after PEG precipitation	< 22%	< 26%	< 3%
ACTH - adrenocorticotropic hormone. CLIA - chemiluminescence. DHEA-S - dehydroepiandrosterone sulfate. DST - dexamethasone suppression test. ECLIA - electrochemiluminescence. MRI - magnetic resonance imaging. n/p - not performed. PEG - polyethylene glycol.			

All three cases were identified between January and June 2022 and presented in the order of their appearance. Written inform consents of all patients were obtained.

The lot of the reagent for ACTH measurement was the same in all cases. The reference intervals are specified in Table 1. The primary clinician who contacted the clinical laboratory in order to express concern about clinically inconsistent results requested repeated ACTH measurements. The interference of the ACTH assay in plasma samples was investigated by a clinical biochemistry specialist. We performed our ACTH measurements using Immulite 2000 xpi (Siemens Healthineers, Erlangen, Germany), which is a solid-phase, two-site sequential chemiluminescent immunometric assay (CLIA) (*2*). For comparison of analytical platforms, we used the Cobas 6000 e601 (Roche Diagnostics, Mannheim, Germany), which is a solid-state electrochemiluminescence immunoassay (ECLIA) (*2*). Blood samples for ACTH measurements were obtained by an educated endocrine nurse after acclimation of the patient to the hospital environment, and prolonged venipuncture was avoided due to the possibility of increasing ACTH concentrations due to stress. The ACTH measurements were conducted using plasma samples obtained from blood sampling tubes with 7.2 mg of K2-ethylenediaminetetraacetic acid (EDTA) (Ayset, Adana, Turkey). As soon as the sample was collected, it was transported to the laboratory on an ice block and thereafter subjected to centrifugation at 2600xg for 10 minutes at 4 °C. In our cases, dilutions were performed manually with distilled water (1:2, 1:4, and 1:8) and measured on the Immulite 2000 XPI. For polyethylene glycol (PEG) procedure, a volume of 300 µl of plasma was combined with an equal volume of 25% PEG (6000 Sigma-Aldrich, Steinheim, Germany) solution. The sample was subjected to centrifugation at 2800xg for 15 minutes after being kept at room temperature for 10 minutes and supernatant portion was used (*10*). The result was multiplied by a factor of two, and the recovery was computed. Heterophilic blocking tube (Part# 3IX762, Scantibodies, Santee, USA) was used and the manufacturer’s instructions were followed.

## Case 1

A 38-year-old woman reported a 8 to 10-kg weight gain in last 6 months and she had a history of hair loss, irritability, fatigue and easy bruisability. Due to the clinical suspicion of CS, the use of exogenous glucocorticoids was thoroughly questioned and excluded. Because of the unsuppressed cortisol secretion and ACTH concentration in the reference interval, pituitary magnetic resonance imaging (MRI) was performed. On pituitary MRI, a 5x5x6 mm left-sided microadenoma was identified, and inferior petrosal sinus sampling (IPSS) was performed (*11*). Sampling was performed with 1 µg/kg corticotropin, following the standardized technique. ACTH concentrations of 10.4 pmol/L, 12.0 pmol/L, and 9.7 pmol/L were measured in the right and left petrosal sinus and peripheral vein samples respectively. The central-to-peripheral ACTH gradients below three (1.1 for the right and 1.2 for the left sinus) ruled out Cushing’s disease (CD) (*11*). In an abdominal MRI, a 30x23x25 mm adenoma in the right adrenal gland was detected. Before screening for ectopic ACTH secretion, the patient’s extremely low dehydroepiandrosterone sulphate (DHEA-S) concentration and adrenal adenoma prompted consideration of ACTH interference and CS coexistence. The plasma ACTH measurements were repeated with the same sample on two different analytical platforms using CLIA (limit of quantification: 1.1 pmol/L) and ECLIA (limit of quantification: 0.2 pmol/L) immunoassays (Table 1). In addition to concentration measured with the ECLIA method being several orders of magnitude lower, serial dilution of plasma samples revealed nonlinearity. Furthermore, recovery of less than 22% following PEG precipitation additionally supported assay interference. The ACTH concentration with CLIA was measured at 8.4 pmol/L following the treatment with heterophile blocking reagent. Following these laboratory findings the patient was diagnosed with CS and unilateral adrenalectomy was performed. The pathology result was compatible with adrenocortical adenoma. Six months after adrenalectomy, the patient had no complaints and was still on physiological dose of hydrocortisone replacement.

## Case 2

A 33-year-old woman presented with currently diagnosed type 2 diabetes mellitus, abdominal striae, a moon face, and a buffalo hump. She had a history of 15 to 20 kg of weight gain in the last 2 years, and she reported that she had irregular menstrual cycles for 2 years. As in the first case, exogenous glucocorticoid use was excluded. No lesion was detected in the pituitary MRI performed due to unsuppressed cortisol secretion and ACTH concentrations in the reference interval. However, a left-sided 3 cm adrenal adenoma was detected on abdominal MRI. Since an ACTH concentration in the reference interval indicates pituitary etiology, before further investigations such as IPSS for the diagnosis of CD, the possibility of ACTH interference was considered because as in the first case the DHEA-S concentration was too low (*11*, *12*). The recovery below 26% after PEG precipitation and ACTH concentration of 2.1 pmol/L obtained following the treatment with heterophile blocking reagent confirmed the interference and allowed the establishment of the CS diagnosis. Unilateral adrenalectomy was performed and the patient continues to be followed up as in remission. Nine months after adrenalectomy, the patient lost 14 kg, her menstrual cycles became regular, oral anti-diabetic medications were stopped and the physiological dose of hydrocortisone replacement was continued.

## Case 3

A 61-year-old female patient was diagnosed with lymhocytic hypophysitis 15 years ago without any symptoms related to mass effects such as headache and visual disturbance. Following the detection of panhypopituitarism, the patient had been receiving physiological doses of hydrocortisone and levothyroxine replacement therapies. While the ACTH concentration, which was measured to adjust the therapy and evaluate the course of the disease, has been within the reference interval since the diagnosis, it was found to be higher than the upper limit of the reference interval with the CLIA immunoassay at the last follow-up visit (Table 1). The patient’s hydrocortisone and levothyroxine replacement doses were unchanged as she had no complaints and her physical examination was normal. On MRI, the pituitary gland was in heterogeneous contrast and no adenoma was detected. As a result of these findings, the possibility of ACTH interference was considered and an assay analysis was performed. Plasma ACTH concentration was approximately 15 times lower when ECLIA was used, serial dilution of plasma samples revealed nonlinearity, and ACTH recovery after PEG precipitation was less than 3% (Table 1). Following the treatment with heterophile blocking reagent, the measured ACTH concentration using CLIA was 69.3 pmol/L. Test results, which are shown in Table 1, suggested ACTH interference. The patient’s current therapy was maintained, and unnecessary diagnostic procedures were avoided.

## Discussion

We presented three cases with ACTH interference that were confirmed with different laboratory methods. According to the diagnostic algorithm for CS, additional examinations, such as pituitary imaging or IPSS, were necessary to ascertain the cause of hypercortisolism in the first patient (*11*). Magnetic resonance imaging is the preferred imaging modality for the detection of pituitary adenomas, including ACTH-secreting ones. According to the guidelines, patients with lesions less than 6 mm on MRI should undergo IPSS, whereas those with lesions measuring 10 mm or larger do not require IPSS. There is a divergence of expert opinions with regards to tumors measuring 6-9 mm. However, a prevailing consensus among the majority of experts suggests that the use of IPSS is recommended (*11*). Inferior petrosal sinus sampling has high diagnostic accuracy for tumor localization to the pituitary gland. In addition, it is possible that a pituitary lesion detected by MRI is an incidental nonfunctioning adenoma or other sellar mass with an ectopic ACTH source (*11*). In the first patient, the IPSS result did not indicate CD, and the detection of ACTH interference precluded further investigation such as whole-body computed tomography or functional imaging modalities for the detection of ectopic ACTH secretion (*11*). In the second case, the diagnosis was made earlier due to awareness of ACTH interference, so the IPSS procedure was avoided. In the first and second patient, the low DHEA-S concentrations raised the suspicion of ACTH interference. It is known that low DHEA-S concentration makes the diagnosis of ACTH-dependent CS very unlikely (*13*). Carafone *et al.* reported the DHEA-S concentration of less than 1.08 μmol/L as potentially indicative of CS. It was also pointed out that autonomous cortisol secretion (ACS) could be diagnosed with higher sensitivity and positive predictive value when serum DHEA-S and plasma ACTH concentrations were used together (*12*). In addition, in case three, ACTH concentration higher than the upper limit of the reference interval may suggest ACTH secreting pituitary adenoma. However, awareness of ACTH interference prevented unnecessary procedures and possible corcerns about pituitary tumor. There are only case reports with ACTH interference in the literature (*2*, *3*, *5*-*9*). The fact that all our cases were identified within six months suggests that, despite being underreported, ACTH interference may occur more frequently than predicted. Similar to the first patient, there are cases in which an invasive procedure, IPSS, had to be performed because the clinical interpretation was complicated as a result of ACTH interference. Moreover, unnecessary pituitary surgery was also performed in one patient (*14*). In literature, in a case with mild ACS, ACTH interference was also observed, making it difficult to follow up the patients even if the patients is subclinical (*8*). According to Ismail *et al.*, interfering antibodies of different types lead to clinically discrepant results in approximately 0.5% of immunoassays, although other studies have reported either a higher or lower incidence (*15*-*17*). Despite this relatively low prevalence, it is essential to recognize the significance of interference and the associated adverse outcomes in clinical management as well as to notify the laboratory specialist if suspicion of the attending clinician arises based on the inconsistent test results.

There is no one specific test that can be considered the “best” for identifying assay interferences (*18*). Various techniques are available, and, for making the choice, their limitations have to be acknowledged. One of these techniques is analyzing the sample with alternative assays using antibody produced in various species, which should typically yield consistent results. There is a high probability of interference if the data obtained on several platforms are found to be markedly inconsistent with one another (*5*, *6*). Most immunoassays use monoclonal or affinity-purified antibodies targeting specific epitopes. Assays from different manufacturers are likely to be directed against distinct epitopes, potentially employing antibodies originating from different animal species (*19*). For instance, the Siemens ACTH reagent uses a polyclonal anti-ACTH antibody, whereas the Roche ACTH reagent utilizes a monoclonal anti-ACTH antibody (*4*). Measurements before and after adding a heterophile antibody blocking reagent may also detect interfering antibodies. Heterophile antibodies, naturally weak antibodies, interfere with the assay noncompetitively (*20*). When incubation with heterophile-blocking tubes causes a considerable change in results, an interferent is likely present. Nonetheless, the absence of change is not proof that there is no interference (*4*). We also used heterophilic antibody blocking tubes to analyze our samples, and in the second case, we detected a decrease in ACTH concentration after using a heterophile blocking tube. On the other hand, the absence of a decrease in the 1st and 3rd cases did not exclude interference. Moreover, serial dilutions method may reveal interferences. In the absence of an interferent, the measured analyte concentration should fall linearly or in parallel as a sample is diluted. An interferent would cause non-linearity and non-parallelism (*21*). Serial dilution of plasma samples of all three patients revealed nonlinearity that suggest assay interference. Unfortunately, the commercial diluent was unavailable in our lab, so distilled water was used for dilutions, which somewhat challenges testing reliability. It is also known that, PEG is a polymer of ethylene oxide that precipitates proteins without denaturing or interfering with them; a 25% PEG-6000 solution precipitates IgG, IgM, and IgA to a maximum of 80% purity (*22*). The majority of reports evaluated the use of PEG precipitation to detect the presence of macroprolactine (*23*). Hence, a diagnostic cut-off concentration for ACTH recovery is uncertain. However, it was assigned as 50% in a study that was conducted by Yener *et al.* (*7*). Values between 3 and 26% in our cases strengthen the possibility of interference.

One of the most important findings from our cases is that evaluating the agreement between test results and clinical data can enhance the identification of interferences. Another important finding is that the plasma ACTH concentration may be in the reference interval and not elevated in cases of interference.

Assay interference with endogenous antibodies, such as heterophile antibodies, human anti-animal antibodies, rheumatoid factor, or other interfering substances such as biotin, has been documented. However, most of the time, the interfering antibody cannot be detected sufficiently (*24*). One of the limitations of our case presentations was the lack of data on endogenous antibodies concentration and the inability to find the substance causing interference.

In conclusion, in clinical practice, increasing awareness of ACTH interference and comprehensive approach in evaluation could allow timely detection and prevent unnecessary invasive diagnostic procedures.

## Data Availability

The datasets generated during and/or analyzed during the current study are available from the corresponding author on reasonable request.
